# Assessment of Antibiotic and Pesticides Residues in Breast Milk of Syrian Refugee Lactating Mothers

**DOI:** 10.3390/toxics7030039

**Published:** 2019-07-31

**Authors:** Nadia Smadi, Adla Jammoul, Nada El Darra

**Affiliations:** 1 Faculty of Heath Sciences, Beirut Arab University, Tarik El Jedidah–Beirut, P.O.Box: 115020, Riad EL Solh, Beirut 1107 2809, Lebanon; 2 Food Department, Lebanese Agricultural Research Institute, Fanar P.O. Box 2611, Beirut 1107 2809, Lebanon

**Keywords:** breast milk, antibiotic residues, pesticide residues, LC-MS/MS, GC-MS/MS

## Abstract

Occupational exposures and current diet are both sources of environmental contaminants that can be transferred in the mother’s body. These chemicals can definitely penetrate to the developing foetus and the nursing infant from contaminated breast milk during the lactation period. Nowadays, one of the special interests is the exposure of new-borns to toxic chemicals such as pesticides and antibiotics reported in human milk due to their potential harms, especially developmental deficits in early childhood. The aim of our current study was to assess the occurrence of pesticide residues and antibiotic residues contamination in breast milk collected from Syrian refugee lactating mothers residing in North Lebanon Camps. A total of 120 breast milk samples (40 in triplicate) were collected from camps in Akkar, North Lebanon using an electrical pump. A survey was administrated to determine socio-demographic characteristics, dietary and smoking habits and medical history of participating lactating mothers. The milk samples were analysed for the presence of antibiotic residues and pesticide residues using liquid and gas chromatography tandem mass spectroscopy (LC-MS/MS) and Gas Chromatography-Tandem Mass Spectrometry (GC-MS/MS). This study reported the absence of antibiotic residues in 96.66% of our samples (*n* = 120) and the presence of pesticides residues in only 5% of our total breast milk sample. Our results considered the breast milk collected from Syrian refugee lactating mothers as safe from chemical contamination. It is worth conducting more studies on other Syrian refugee camps to test the effect of camp living conditions on breast milk safety.

## 1. Introduction

Breastfeeding is admitted to present numerous beneficial health effects and it is vastly considered as the best suitable food for the baby [1]. The World Health Organization recommends to exclusively breastfeed for six months after birth [2]. However, the European recommendation varies among countries between four and six of exclusive breastfeeding [3].

In Lebanon and Syria, two Middle Eastern Arab countries sharing almost the same socio-demographic characteristics including religion and language, a percentage of 10% was shown for an exclusive breastfeeding rate at six months [4]. According to the United Nations Children Fund [5], the average of breastfed Syrian infants under six months of age was found to be 43%. The absence of support for breastfeeding by social workers and health care providers were considered the main reasons for low prevalence of exclusive breastfeeding. It could be also due to psychological reasons such as mother’s emotional stress and the perceived breast-milk inadequacy [6].

Breast milk is an important source of energy and nutrients for children’s health [7]. However, many studies have been conducted to assess the chemical contaminants in breast milk associated with its health effects on the infant and the mother. This contamination could occur due the exposure of the nursing mother on a daily basis to chemical pollution of the environment. These environmental chemicals are released from several basic activities and different sources such as water, air, food and manufactured products [8].

Few research studies have reported the presence of antibiotic and pesticide residues in breast milk. It is worth mentioning that studies screening antibiotic residues are rare on the international scale. The latest study conducted in Turkey [9], detected quinolone and beta-lactam residues in human milk samples, with an occurrence of 8/34 (23.5%) and 29/34 (85.2%), respectively. It is to be noted that the lactating mothers enrolled in this study do not have antibiotic history. Antibiotic residues in human milk were thought to derive from the mothers’ food such as chicken, meat and dairy products [10].

Additionally, some studies have screened the presence of pesticide residues in breast milk especially organochlorine pesticides [11,12,13,14,15]. The most important study was carried out in China where a wide range of concentrations for 23 organochlorine pesticide (OCP) compounds were detected in 24 pooled samples of breast milk. The dichlorodiphenyltrichloroethane (DDT) contamination in breast milk was the highest with a mean value 582.8 ± 362.7 ng/g. The explanation for such high contamination was mainly from the mother’s dietary intake since positive correlation was observed between concentration of DDT in human milk and consumption of animal origin food in the Chinese population [16].

However, to our knowledge no studies have been conducted on Syrian refugee lactating mothers residing in camps especially where the living and environmental conditions are very poor. Moreover, our study is the first conduced in Lebanon, screening antibiotics and pesticide residues in breast milk. Thus, the aim of this study was to assess the occurrence of antibiotic and pesticide residues in the breast milk of Syrian refugee lactating women residing in Lebanon camps. It investigated as well the socio-demographic and nutritional factors associated with milk contamination.

## 2. Materials and Methods

### 2.1. Study Population and Area

This cross-sectional study was conducted in Syrian refugee camps located in Lebanon, North Lebanon city—Akkar camps. The participants in this study were lactating Syrian refugee mothers residing in camps. In total, 40 women were randomly recruited and invited to participate in the study by providing mature breast milk samples.

### 2.2. Ethical Approval

The study protocol and ethics of this study were approved by the institutional review board (IRB) code: 2019H-0087-HS-M-0325 (17 January 2019), of Beirut Arab University prior to approaching the participants and a written, signed informed consent was obtained from all participants. All women who volunteered to participate in this study were informed about the purpose of the study and agreed to be part of it.

### 2.3. Questionnaire

A questionnaire was used to collect data about the socio-demographic characteristics of participating lactating mothers. The questionnaire was adopted from different studies [14,16,17,18,19,20]. It consisted of eight subsections collected from different sources, addressing general information about the lactating mothers and the new-borns, dietary habits of lactating mothers, smoking status, geographic characteristics, pesticides spraying, medical history and supplements intake as well as the drinking water intake.

The questionnaire was translated into Arabic to ensure accuracy of the translation, back translation from Arabic to English was conducted by another translator. Both English versions were the same. The Arabic version of the questionnaire was pilot-tested on a sample of 30 mothers. The questionnaire was administered twice within a period of two weeks between each sitting. To determine test–retest reliability, a paired *t*-test was performed to compare mean scores at T_1_ (before) and T_2_ (after two weeks). Pearson’s correlation was calculated between T_1_ and T_2_, with a consistency interclass correlation (ICC) [21]. The values of ICC were considered excellent if >0.75 [22].

### 2.4. Sample Collection

During December 2018 and February 2019, 50 nursing mothers were contacted but only 45 agreed to answer the survey and participated in our study. The 45 nursing mothers that were visited for the survey were contacted again and only 40 of them agreed to donate breast milk by triplicate over three weeks.

Breast milk collection was done in the morning an hour after the previous breastfeeding. Samples (25–40 mL) were collected using a sterile, single-use electrical pump (Chico, Mod.06836, China). A polypropylene pump container was sterilized with 70% alcohol solution to prevent cross contamination between samples. Each sample was placed into a separate 100 mL dark glass (to preserve the samples from light), numbered then transported on ice (at 4 °C) to the laboratory, where they were transferred into urine cups, numbered and stored at −20 °C until analysis.

### 2.5. Chemicals and Reagents

The reagents used were of analytical grade. The antibiotic standards belonged to three different families: sulphonamides (sulfamethazine), tetracyclines (tetracycline, oxytetracycline) and beta-lactam antibiotics (ampicillin); in addition, 161 kinds of pesticide were purchased from Sigma-Aldrich (St. Louis, MO, USA). The standards used present high purity grades (>99%). Individual stock solutions were prepared at 1000 µg/L in acetonitrile and stored at −20 °C.

The working standard solutions of a concentration of 10 µg/L each were prepared as dilution of the stock solution in water/methanol (50:50, *v*/*v*). The working standard solutions were stored at −20 °C. HPLC-grade water, HPLC-grade acetonitrile, and magnesium sulphate (MgSO_4_) were also supplied by Sigma-Aldrich.

### 2.6. Sample Extraction for Antibiotics Residues Analysis

Using liquid chromatography–mass spectrometry, a multi-class method was developed for identifying and quantifying three antibiotics belonging to two different chemical classes (tetracyclines and beta-lactams). Afterwards, this method was optimized for the detection of antibiotics in breast milk.

100 μL from 1 % formic acid was added to a 2 mL of breast milk then vortexed for 60 s and kept in the dark for 10 min. Then 500 μL of EDTA (0.5%) was added and shaken vigorously for 1 min. Next, 8 mL TCA (5%) was added then shaken for 10 min before subjecting the tubes to centrifugation at 5000 rpm at 4 °C for 10 min. The supernatant obtained was then subjected to filtration through a 0.45 μm polyvinylidene fluoride (PVDF) filter for further liquid chromatography tandem mass spectroscopy (LC-MS/MS) analysis.

### 2.7. Sample Extraction for Pesticide Residues Analysis: QuEChERS Extraction

To ensure a quick and easy sample treatment, a modified QuEChERS (quick, easy, cheap, effective, rugged and safe) approach, originally developed as a powerful sample preparation, was used to analyse hundreds of pesticides in different kinds of food.

Acetonitrile (10 mL) was added to 10 mL of breast milk and shaken vigorously before adding magnesium sulphate MgSO_4_ (4 g), sodium chloride NaCl (1 g), sodium dibasic citrate (1 g) and sodium tribasic citrate (0.5 g) and then shaken for 3 min. The tubes were then subjected to centrifugation at 5000 rpm at 4 °C for 5 min.

The aqueous supernatant (5 mL) was mixed with dispersive Enhanced Matrix Removal (EMR) dissolved in 5 mL water then shaken vigorously for 5 min. Another centrifugation was conducted at 5000 rpm at 4 °C for 5 min.

The aqueous supernatant (5 mL) obtained was transferred in a new Polish EMR Lipid (MgSO4 and NaCl tube, then shaken vigorously for 3 min before being centrifuged at 5000 rpm at 4 °C for 5 min. Afterwards, a filtration was made through a 0.22 μm polyvinylidene fluoride (PVDF) filter for further LC-MS/MS and GC/MSMS analysis.

### 2.8. LC-MS/MS Equipment

LC-MS/MS analyses was conducted using an Agilent 6430 LC/MS (Agilent technologies, Santa Clara, USA) for antibiotic and pesticide residues detection, and LC-MS/MS analyses were performed on LC-NexeraX2 Shimadzu 8045 LC/MS (Kyoto, Japan). The mass spectrometer was operated with an electron spray ionisation (ESI) in multiple reaction monitoring (MRM) mode, ionspray voltage 4 kV, nitrogen for desolvation and dried gas 11 L/min.

The quantification of the three antibiotics families and 161 kinds of pesticide in 120 breast milk samples was done through measuring peak areas in the multiple reaction monitoring chromatogram, and comparing them with the relevant matrix-matched calibration curves. A calibration curve ranging between 1 μg/L and 500 µg/L was carried out to verify linearity.

The performance of the analytical method was evaluated by checking the identification criteria for the presence of two transitions at the same retention time, the signal to noise ratio ≥ 10, the relative retention time of the analyte within a tolerance of 2.5% and the relative ion intensities ratio within a tolerance defined by the EU commission decision 2002/657/EC. The MRM parameters are shown in Table 1.

### 2.9. LC/MS/MS Parameters

The separation of the pesticide residues was performed using a C18 analytical column (zorbax 2.1 mm inner diameter × 150 mm length, 3.5 μm particle size; Agilent technologies, Santa Clara, CA, USA) and a C18 analytical column (Shim-pack GIST 2.1 mm inner diameter × 100 mm length, 3 μm particle size; Japan) for antibiotic residues separation. The separation of pesticides was accomplished at 40 °C. The flow rate and injection volume were 0.4 mL/min and 10 µL, respectively. The mobile phases used were (A) 5 mM ammonium acetate and 0.1% formic acid in water, and (B) 5 mM ammonium acetate in methanol. The gradient elution program started with 100% A for 5 min, then decreased to 50% for 13 min, then to 0% for 2 min and returned back to the initial conditions for 5 min. The final run time of the method was 29 min.

The separation of sulfamides, tetracyclines and beta-lactam was accomplished at 40 °C. The flow rate and injection volume were 0.3 mL/min and 10 µL, respectively. The mobile phases used were (A) 5 mM ammonium acetate and 0.1% formic acid in water, and (B) 5 mM ammonium acetate in methanol. The gradient elution program was as follow: B (40%) (2 min), B (90%) (9 min), B (10%) (9 min and 1 s), the final run time of the method was 13 min.

### 2.10. GC/MSMS Parameters

GC-MS/MS analyses were carried out with an Agilent 7890A GC equipped with 7693 Agilent auto-sampler and 7000C triple quadrupole GC/MS system. The separation was performed on a HP-5MS Agilent technologies (Santa Clara, CA, USA), 0.25 mm × 30 m, 0.25 μm, and helium (purity 99.996%) was used as a carrier gas at a constant pressure of 11 psi. The inlet temperature was set at 250 °C; the mode of inlet was splitless; the injection volume was 1 μL. The column temperature program was as follows: the initial temperature was maintained at 70 °C for 1 min, increased to 160 °C at a rate of 50 °C/min, raised to 200 °C at 2 °C/min, and then at 16 °C/min up to 280 °C, and held there for 7.2 min. The total run time was 35 min. The mass spectrometer was operated with an electron impact (EI) source in multiple reaction monitoring (MRM) mode. The electron energy was 70 eV, and the source, transfer line and quadrupole temperatures were set at 280 °C and 150 °C, respectively. In order to prevent instrument damage, the solvent delay was set at 4.5 min.

### 2.11. Recovery Test

In-house validation was performed by fortifying the blank matrix at a level of 50 µg/L. The extraction was performed by the methods described in the Section 2.6 and 2.7. The spiked and blank samples were then analysed by LCMS/MS. Recovery was calculated by comparing the analysed concentrations with spiked concentrations.

## 3. Results and Discussion

### 3.1. Validation of the Arabic Version of the Questionnaire

The results of test–retest reliability are presented in Table 2. The paired *t*-test showed that the mean scores of the Arabic questionnaire did not vary significantly between T_1_ and T_2_. A statistically significant inter-item correlation, *p* < 0.05, was noted between T_1_ and T_2_. An excellent consistency was also noted, with an ICC varying between 0.998 and 1.00 [22].

### 3.2. Survey Results

The results of the questionnaire filled by 40 Syrian refugee nursing mothers is presented in Table 3. All the participants had an age below 30 years. The number of years of residency in North Lebanon camps for all lactating mothers was between one year and five years. All the mothers (100%) had an educational degree below secondary and they were not employed.

While assessing the smoking habits, it was shown that 100% of nursing mothers had never smoked before or after pregnancy since Syrian culture inhibits women from smoking, but they were randomly exposed to second hand smoking from their husbands. Lactating mothers were also asked about their supplement intake, 100% indicated that they were taking vitamins and iron supplements during their pregnancy period, however they stopped consuming these supplements after their child’s birth in order to save money.

When questioned on their dietary habits, it was clearly noticed that 100% of the participants never consumed fish and sea food due to poverty. The consumption of meat and poultry was also negligible for the same reason. With respect to their location of residence, 100% were living near a waste disposal site and cultivation activity since the camp was located in an agricultural area.

### 3.3. Assessment of Antibiotic Residues in Breast Milk

#### 3.3.1. LC-MS/MS Method Performance for Antibiotic Residues

An in-house method was verified as per the criteria specified in EU commission decision 2002/675/EC to quantify antibiotics residues. The validation parameters were fixed by spiking blank powdered milk at a level of 50 ng/g. The measured parameters were specificity, linear range, repeatability, reproducibility, accuracy and limit of quantification (LOQ).

**Figure 1:**
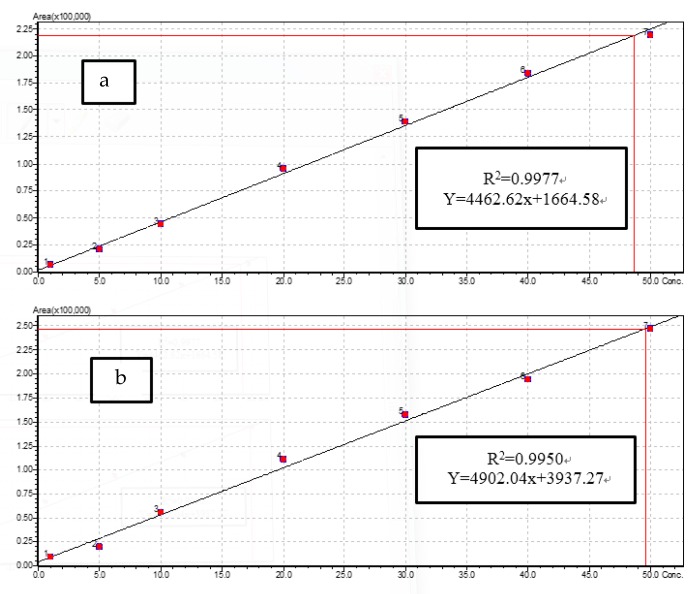

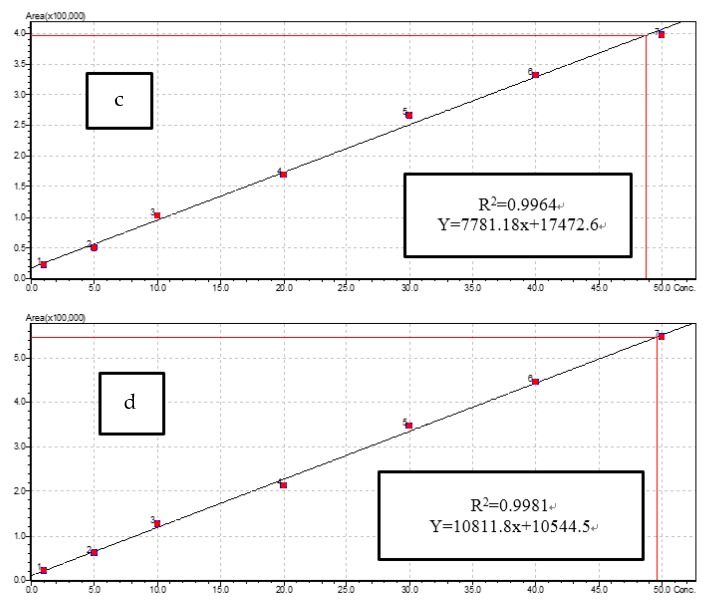
Calibration curves for (**a**) oxytetracycline, (**b**) tetracycline, (**c**) ampicillin and (**d**) sulfamethazine 1, 5, 10, 20, 30, 40 and 50 µg/L.

The antibiotic residue chromatograms of the reference standards used as well as the calibration curves are shown in Figure 1. The calibration curves were created from six matrix-calibration standards which were injected in each batch in the range of 1 to 50 ng/g. The calibration curves showed good linearity, characterized by a high correlation coefficient (*R*^2^ > 0.99).

The precision of the method was determined using the spiked standards at the 50 µg/L level. The results for repeatability ranged from 5 and 18.8% (Table 4). The limit of quantification is considered as the lowest quantified level with S/N ≥ 10 in presence of the two transitions at the same retention time. The LOQ was calculated to be 2 μg/kg for all tested antibiotics. The mean recoveries of the residues for the spiked samples ranged between 97% and 108% (Table 4). These values presented high mean recoveries within the acceptable range (80%–120%) except for ampicillin, which presented a low mean recovery, although it was still higher than 50%. These values were within the acceptable ranges (50%–120%) recommended by AOAC, 2002 [23].

#### 3.3.2. Occurrence of Antibiotic Residues in Breast Milk Samples

The method developed was applied to the determination of four antibiotics from three different chemical classes (sulphonamides, tetracyclines, and beta-lactams) in 120 breast milk samples collected from 40 lactating mothers residing in Lebanon’s camp. In order to validate the results, an internal quality control was carried out on every batch of samples. Moreover, the retention time, quantification and confirmation transitions and relative ion intensities of the detected ion of the human milk samples were compared to those of the corresponding calibration standards in the same batch to identify the detected analytes.

While assessing the antibiotic residues in the breast milk samples (Table 5), the results showed that only 3.33% of the samples were contaminated with antibiotic residues. It is to be noted that none of the samples contained more than one antibiotic residues. The antibiotic residue contaminating the four samples was oxytetracycline. Of the samples, 96.67% presented a total absence of antibiotic residues. This result is well correlated with the survey, that shows that all the mothers did not receive antibiotics during pregnancy nor lactation period. Additionally, the survey results showed that 100% of the nursing mothers do not consume meat and chicken, that are known to be sources of antibiotic residues, due to the misuse of antibiotics in farms [10,24].

#### 3.3.3. Mean Concentrations of Antibiotic Residues for the Different Families in Breast Milk Samples

Table 5 represents the summary of multi-antibiotic residues occurrence in 120 human milk samples. The assessment of the sulphonamides, beta-lactam families and tetracyclines showed that three antibiotic residues were not detected in all milk samples, i.e., tetracyclines, sulfamethazine and ampicillin. However, as mentioned before, oxytetracycline was detected in four samples (3.3%). These four samples belong to three mothers; one of them reported one antibiotic residue concentration between the LOQ (= 2 µg/L) and LOD (= LOQ/3 = 2/3 = 0.66µg/L) followed by a second concentration higher than the LOQ in the week after. The other two samples exceeded the LOQ.

Hence, the percentage of positive samples (>LOQ = 2 µg/L) is 2.5%, since the fourth sample presented a mean of 0.64 µg/L which is lower than LOQ and LOD.

The mother that reported the highest value of oxytetracycline in her breast milk (6 µg/L) was the only mother that consumed milk on a daily basis. The mean value of oxytetracycline (5.04 µg/L) is 2.5 times higher than the LOQ (2 µg/L). The low oxytetracycline concentration in only three breast milk samples is derived from the nursing mother’s food since they did not use any kind of antibiotics after pregnancy. However antibiotic residues can be found in dairy products since antibiotics are usually used in farms of dairy cattle for the treatment of diseases such as mastitis [25]. Probably the source of oxytetracycline is from nursing mothers’ dairy product intake because our survey findings indicate the contaminated breast milk samples belong to 15% of mothers consuming dairy products twice per week. The presence of oxytetracycline in dairy products, could be due to the misuse of this antibiotic in farms and lack of abidance to the recommended withdrawal times [26].

### 3.4. Assessment of Pesticide Residues in Breast Milk

#### 3.4.1. LC-MS/MS Method Performance for Pesticide Residuess

**Figure 2:**
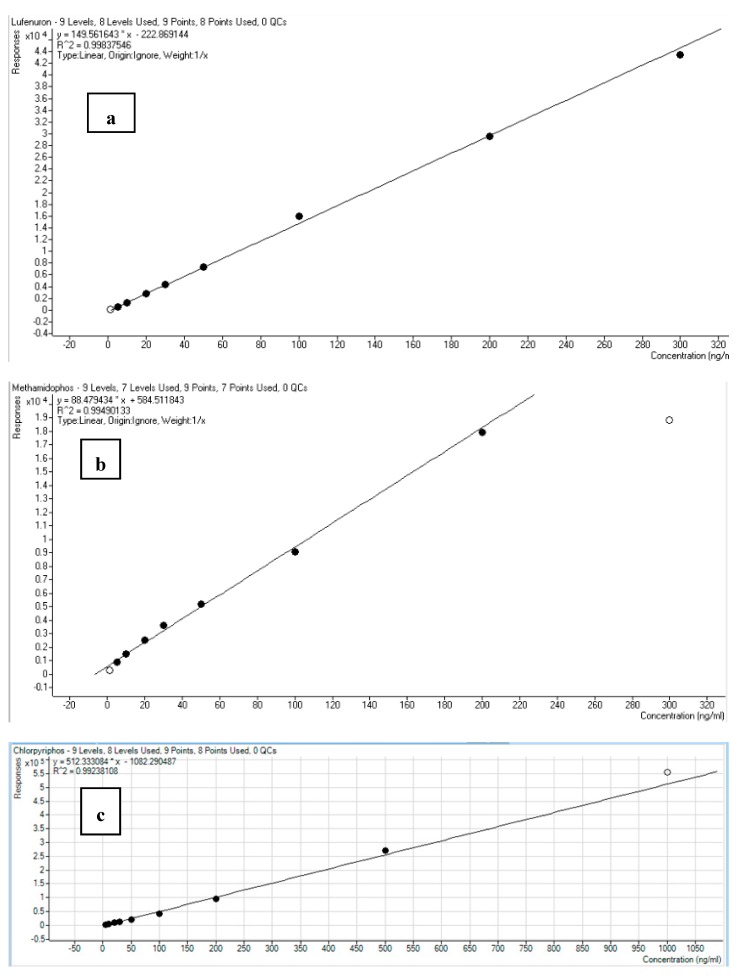
LC standard calibration curves for (**a**) lufeneron, (**b**) methamidophos and (**c**) chlorpyriphos at 5, 10, 20, 30, 50, 100, 200, 500 µg/L.

The pesticide residue chromatograms of the reference standards used as well as the calibration curves are shown in Figure 2. The calibration curves were created from nine matrix-calibration standards which were injected in each batch in the range from 1 to 500 ng/mL. The calibration curves showed good linearity, characterized by a high correlation coefficient (*R*^2^ > 0.99).

The precision of the method was determined using two spiked levels of 0.05 and 0.1 mg/kg. The limit of quantification was considered as the lowest quantified level with S/N ≥ 3 in presence of the two transitions at the same retention time. The LOQ was calculated to be 5 μg/kg for all tested pesticides. The mean recoveries of the residues for the spiked samples was between the acceptable range (80%–120%).

#### 3.4.2. Occurrence of Pesticide Residues in Breast Milk Samples

While assessing the pesticide residues in the breast milk samples, the results showed that only 5% of the samples were contaminated with pesticide residues. The samples 95% were non-contaminated. It is to be noted that none of the samples contained more than one pesticide residue.

#### 3.4.3. Mean Concentrations of Pesticide Residues in Breast Milk Samples

Table 6 represents the summary of multi-pesticide residues occurrence in 120 human milk samples. The assessment of the 161 pesticides showed that only three pesticide residues were detected in six samples, lufeneron, methamidophos and chlorpyriphos. Hence, the percentage of positive samples (>LOQ = 5 µg/L) was 4.16%. Only four samples were contaminated with lufeneron, the fifth sample was contaminated with methamidophos and the final sample was contaminated with chlorpyriphos with a concentration of 12.32 µg/L. The recovery tests results were at one level (10 µg/L) for methamidophos (86.5%), chlorpyriphos (103.6%) and lufeneron (105.2%).

While assessing the pesticide residues in breast milk, the percentage of positive samples was only 5% in which methamidophos presented the highest concentration (13.1927 µg/L) in one sample only. The only mean value that exceeded the LOQ (5 µg/L) was reported for lufeneron pesticide (5.874 µg/L). The present results can be explained by nursing mother exposure to pesticides from fruit and vegetable intake [27], since the percentage of mothers consuming fruit and vegetables twice per week were 55% and 35% respectively, to the contrary of other studies conducted in several countries where the percentage of pesticide contamination in breast milk is high [14].

## 4. Conclusions

The contamination of breast milk is a critical problem since it affects the health of both the mother and her infant. In the last few decades, numerous studies reported chemical contamination in breast milk, especially pesticide residues, but antibiotic residue studies on breast milk remain rare. These contaminations are usually associated with socio-demographic status and dietary habits of nursing mothers. This study conducted for the first time, in Syrian refugee camps, presented an absence of antibiotic residues in the majority of the samples and the presence of pesticide residues in only 5% of our total breast milk sample. These findings consider the breast milk collected from Syrian refugee lactating mothers safe from the chemical contaminants screened. This could be due to the poor living and environmental conditions in the camp. It is worth conducting more studies on other Syrian refugee camps to test the effect of the camp living conditions on breast milk safety.

## Figures and Tables

**Table 1:** Multiple reactions monitoring (MRM) acquisition condition for each antibiotic used.

Antibiotic Family	Antibiotic	Precursor Ion (*m*/*z*)	Collision Energy for Precursor Ion (eV)	Product Ion (*m*/*z*)	Collision Energy for Production Ion (eV)	Cone Voltage (V)	Retention Time (min)
Sulphonamides	Sulfamethazine	279.0 > 186.1	17	279.0 > 184.0	23	Default	5.012
Tetracyclines	Tetracycline	445.1 > 410.25	20	445.1 > 427.15	12		4.886
	Oxytetracycline	461.1 > 443.3	14	461.1 > 426.15	18		4.934
Beta-Lactam	Ampicillin	350.3 > 106.05	20	350.3 > 159.9	13		4.944

**Table 2:** Mean and standard deviations, Pearson’s correlation coefficients and intraclass correlation for the scores of the Arabic and Arabic versions (*n* = 30).

Scale	Mean Scores			Correlation between Scores at T1 and T2	Intraclass Correlation (ICC)	
	Arabic T_1_	Arabic T_2_	Paired *t*-Test			
	Mean ± SD		*p*-Value	Correlation Coefficient	ICC	95% CI
Dietary Habits of Lactating Mothers	71.2 ± 10.21	71.43 ± 10.23	0.032	0.998	0.998	0.996–0.999
Smoking Status	5.16 ± 0.94	5.14 ± 0.92	0.000	0.999	0.999	0.998–1.000
Demographic Characteristics	3.33 ± 0.711	3.32 ± 0.743	0.001	1.000	1.000	1.000
Pesticides	2.866 ± 0.860	2.867 ± 0.862	0.000	1.000	1.000	1.000
Medical History and Supplements Intake	6.566 ± 1.381	6.565 ± 1.380	0.000	0.999	0.999	0.998–1.000

**Table 3:** Survey results of participating mothers.

Characteristics	Frequency	Percent
Residence years in Tripoli Lebanon camps (1–5 years)	40	100
Age (<30 years)	40	100
Gestational Age (= 9 months)	40	100
Lactation time (>= 120 days)	40	100
Level of education (Below secondary)	40	100
Occupation Not employed (Housekeeper)	40	100
Number of newborn (=1 child)	40	100
Infant Gender		
Male	16	40
Female	24	60
Age of newborn		
=< 5months	11	27.5
>5 months	29	72.5
Birth weight (g)		
=< 3.5 kg	39	97.5
> 3.5kg	1	2.5
Irregular newborn Sleep pattern	40	100
Colic Crying of newborn	40	100
Fish intake		
Never	40	100
Sea food intake		
Never	40	100
Cereals intake		
Twice a week	39	97.5
Daily	1	2.5
Potatoes intake		
Once a week	7	17.5
Twice a week	2	5.0
More than twice a week	18	45
Daily	13	32.5
Fresh Vegetables intake		
Once a week	26	65
Twice a week	14	35
Milk intake		
Never	39	97.5
Daily	1	2.5
Dairy Product intake		
Never	11	27.5
Once a week	19	47.5
> once a week	4	10
Twice a week	6	15
Pasta intake		
Once a week	15	37.5
More than Once a week	5.0	5.0
Twice a week	37.5	37.5
More than Twice a week	20.0	20.0
Rice intake		
More than Twice a week	10	25.0
Daily	30	75.0
Grains		
Once a week	4	10.0
Twice a week	13	32.5
More than Twice a week	23	57.5
Soft Drink intake		
Never	12	30.0
Once a week	20	50.0
More than Once a week	2	5.0
Twice a week	6	15.0
Jam intake		
Never	27	67.5
Once a week	13	32.5
Coffee intake		
Never	23	57.5
Once a week	9	22.5
Twice a week	8	20.0
Tea intake		
Once a week	1	2.5
More than Once a week	1	2.5
Twice a week	11	27.5
More than Twice a week	12	30.0
Daily	15	37.5
Fruits intake		
Once a week	22	55.0
Twice a week	18	45.0
Salty Snack intake		
Never	19	47.5
Once a week	21	52.5
Chocolate intake		
Never	21	52.5
Once a week	17	42.5
Twice a week	2	5.0
Meat and Poultry intake		
Never	40	100
Eggs intake		
Never	16	40.0
Once a week	13	32.5
More than Once a week	6	15.0
Twice a week	5	12.5
Beverages		
Never	11	27.5
Once a week	17	42.5
More than Once a week	3	7.5
Twice a week	9	22.5
Smoking status		
Before pregnancy	40	100
No	40	100
During pregnancy	40	100
No		
Random smoke exposure		
Yes		
Nearby Waste Disposal		
Yes	40	100
Nearby Cultivation Activity		
Yes	40	100
Spray indoor to prevent mosquitoes		
Yes	40	100
Spray outdoor with pesticides		
Yes	40	100
Vitamin supplement in pregnancy		
Yes	40	100
Iron supplement in pregnancy		
Yes	40	100
Vitamin_supplement_in_postpartum_2_months		
No	40	100
Iron_supplement_in_postpartum_2_months		
No	40	100
Antibiotic intake in pregnancy		
No	40	100
Antibiotic intake after pregnancy		
No	40	100
Anemia at any time		
Yes	40	100
Drinking water		
Well artesian water	40	100
Water bottles intake		
1–2 bottle	40	100

**Table 4:** Results of in-house verification of the LC-MS/MS method for the antibiotics considered in this study, with the standard deviation (STDEV), relative standard deviation of repeatability (RSD) and limits of quantification (LOQ).

Antibiotic Family	Antibiotic	Spiking Level (50 µg/L)	Mean Recoveries (%)	STDEV	RSD (%)	LOQ (µg/L)
Sulphonamides	Sulfamethazine	49.075	98.15	5.72	5.82	2 µg/L
Tetracyclines	Tetracycline	53.85	107.71	6.96	6.46	
	Oxytetracycline	48.664	97.328	18.22	18.72	
Beta-lactam	Ampicillin	26.475	52.95	0.627	1.18	

**Table 5:** Occurrence of sulphonamides, tetracyclines, and β-lactams in the 120 breast milk samples.

Breast Milk Samples (*n* = 120)	Sulphonamides	Tetracyclines		β-Lactams
	Sulfamethazine	Tetracycline	Oxytetracycline	Ampicillin
Mean * (µg/L)	0	0	5.04	0
min (µg/L)	0	0	0	0
max (µg/L)	0	0	6	0
*n* positive	0	0	3	0
% positive	0	0	2.5	0

* mean value of four contaminated samples.

**Table 6:** Occurrence in the 120 breast milk samples of lufeneron, methamidophos and chlorpyriphos.

Breast Milk Samples (*n* = 120)	Lufeneron	Methamidophos	Chlorpyriphos
Mean * (µg/L)	5.8754	2.198	2.05
min (µg/L)	5.1208	0	0
max (µg/L)	12.0447	13.1927	12.32
*n* positive	4	1	1
% positive	3.33	0.83	0.83

* mean values of six contaminated samples.
